# The development of lived experience-centered word clouds to support research uncertainty gathering in degenerative cervical myelopathy: results from an engagement process and protocol for their evaluation, via a nested randomized controlled trial

**DOI:** 10.1186/s13063-021-05349-8

**Published:** 2021-06-25

**Authors:** Benjamin M. Davies, Oliver D. Mowforth, Danyal Z. Khan, Xiaoyu Yang, Sybil R. L. Stacpoole, Olesja Hazenbiller, Toto Gronlund, Lindsay Tetreault, Sukhvinder Kalsi-Ryan, Michelle L. Starkey, Iwan Sadler, Ellen Sarewitz, Delphine Houlton, Julia Carter, Evangeline Howard, Vafa Rahimi-Movaghar, James D. Guest, Bizhan Aarabi, Brian K. Kwon, Shekar N. Kurpad, James Harrop, Jefferson R. Wilson, Robert Grossman, Emma K. Smith, Angus G.K. McNair, Michael G. Fehlings, Mark R. N. Kotter

**Affiliations:** 1 Academic Neurosurgery Unit & Anne McLaren Laboratory of Regenerative Medicine, Department of Clinical Neurosurgery, University of Cambridge, Cambridge, UK; 2 Department of Clinical Neuroscience, Peterborough Hospital, North West Anglia NHS Foundation Trust and Cambridge University Hospitals NHS Foundation Trust, Addenbrooke’s Hospital, Cambridge, UK; 3 AOSpine Spinal Cord Injury Knowledge Forum, Davos, Switzerland; 4 James Lind Alliance, National Institute for Health Research, Southampton, UK; 5 Division of Neurosurgery, Toronto Western Hospital, University Health Network, University of Toronto, Toronto, Ontario Canada; 6 Toronto Rehabilitation Institute-LC, University Health Network, Toronto, Ontario Canada; 7 Myelopathy.org (Registered Charity England and Wales, No 1178673), Cambridge, UK; 8 The Goffin Consultancy, Goffin Consultancy Ltd, Riding House, Bossingham Road, Stelling Minnis, Canterbury, CT4 6AZ UK; 9 US Person with DCM Representative – CSU, Bakersfield, CA USA; 10 Academic Department of Neurological Surgery, Sina Trauma and Surgery Research Center, Tehran University of Medical Sciences, Tehran, Iran; 11 Department of Neurological Surgery, Miller School of Medicine, University of Miami, Miami, FL USA; 12 Division of Neurosurgery, Shock Trauma, University of Maryland, Baltimore, MD USA; 13 Division of Spine Surgery, Vancouver General Hospital, University of British Columbia, Vancouver, British Columbia Canada; 14 Department of Neurosurgery, Medical College of Wisconsin, Milwaukee, WI USA; 15 Division of Neurosurgery, Thomas Jefferson University Hospital, Philadelphia, PA USA; 16 Division of Neurosurgery, Department of Surgery, University of Toronto, Toronto, Ontario Canada; 17 Division of Neurosurgery, Houston Methodist Hospital, Houston, TX USA; 18 School of General Practice, NHS Health Education East of England, Cambridge, UK; 19 Center for Surgical Research, Bristol Medical School: Population Health Sciences, University of Bristol, Bristol, UK; 20 Wellcome Trust & MRC Cambridge Stem Cell Institute, Cambridge, UK

**Keywords:** Cervical, Myelopathy, Word cloud, OPLL, Spondylosis, Disc herniation, Cervical stenosis, Protocol, Outcome, Dataset, Core outcomes in effectiveness trials (COMET), James Lind Alliance (JLA), Research priorities, Delphi, Consensus, Audit, Surveillance, Common data elements (CDE)

## Abstract

**Objectives:**

AO Spine REsearch objectives and Common Data Elements for Degenerative Cervical Myelopathy [RECODE-DCM] is a multi-stakeholder consensus process aiming to promote research efficiency in DCM. It aims to establish the top 10 research uncertainties, through a James Lind Alliance Priority Setting Partnership [PSP]. Through a consensus process, research questions are generated and ranked. The inclusion of people with cervical myelopathy [PwCM] is central to the process. We hypothesized that presenting PwCM experience through word cloud generation would stimulate other key stakeholders to generate research questions better aligned with PwCM needs. This protocol outlines our plans to evaluate this as a nested methodological study within our PSP.

**Methods:**

An online poll asked PwCM to submit and vote on words associated with aspects of DCM. After review, a refined word list was re-polled for voting and word submission. Word clouds were generated and an implementation plan for AO Spine RECODE-DCM PSP surveys was subsequently developed.

**Results:**

Seventy-nine terms were submitted after the first poll. Eighty-seven refined words were then re-polled (which added a further 39 words). Four word clouds were generated under the categories of diagnosis, management, long-term effects, and other. A 1:1 block randomization protocol to assess word cloud impact on the number and relevance of PSP research questions was generated.

**Conclusions:**

We have shown it is feasible to work with PwCM to generate a tool for the AO Spine RECODE-DCM nested methodological study. Once the survey stage is completed, we will be able to evaluate the impact of the word clouds. Further research will be needed to assess the value of any impact in terms of stimulating a more creative research agenda.

**Supplementary Information:**

The online version contains supplementary material available at 10.1186/s13063-021-05349-8.

## Introduction

Degenerative cervical myelopathy [DCM] is the most common cause of spinal cord dysfunction worldwide [1]. It arises when arthritic changes in the cervical spine lead to its narrowing - causing compression injury to the cervical spinal cord. Currently, despite the best available treatment [2], many people with DCM will be left with life-changing disabilities [3] and some of the worst quality of life scores of chronic diseases [4].

AO Spine REsearch objectives and Common Data Elements for Degenerative Cervical Myelopathy [RECODE-DCM] is a multi-stakeholder consensus process which ultimately aims to accelerate research progress, through the formation of recommendations that improve research efficiency [5]. It combines several consensus initiatives, including to confirm the definition of DCM, to establish the top 10 research uncertainties, and to establish a minimum critical dataset for clinical research, care, and audit.

The process to establish research uncertainties is supported by organizations such as the James Lind Alliance [JLA] [6] as a Priority Setting Partnership [PSP]. JLA methodology starts by seeking research suggestions from patients, family, caregivers, and front-line healthcare professionals. Commonly this has been delivered using an electronic survey, with sub-sections adapted to the condition and scope, to stimulate ideas. For AO Spine RECODE-DCM, it was established that these sub-sections would include diagnosis, treatment, long-term management, and other issues [5].

A major driver of inefficiency in health research is proposed to be the exclusion of end-users (e.g., people with cervical myelopathy [PwCM]) from participation in research design [7, 8]. Their involvement is central to impactful results [9–11]. While this is recognized in the PSP by their participation in the surveys, we hypothesized that their experience could also be used to stimulate other stakeholders to generate research questions aligned with this user group. Their input should be helpful as one of the challenges for a DCM PSP is that a diversity of healthcare professionals are involved in DCM care, but generally, it forms a minority of their practice or is confined to a short stage of the disease, e.g., diagnosis [12, 13]. This lack of information and the narrow foci of each practitioner’s role have been proposed to hamper modern clinical research creativity [14].

Word clouds are a tool which enables qualitative data to be displayed; the importance or frequency is assigned by word size or orientation. In the medical literature, word clouds are mainly used to report qualitative patient interview data [15], although further afield they have been used to stimulate creativity [16, 17].

This article describes the generation of word clouds of terms suggested by individuals with DCM, for diagnosis, treatment, long-term management, and other aspects of DCM, so that they can be used in the PSP survey. The article also outlines how word clouds will be nested within AO Spine RECODE-DCM, in order to evaluate their impact on responses in the online survey. This will give insight into their role and value to stimulate a creative research agenda from respondents to surveys about research priorities.

## Methods

The word clouds were generated working with Myelopathy Support, an online peer-to-peer support community for individuals with DCM and their caregivers. Ethical approval has not been required for the involvement of the Myelopathy Support members as their role is to help to develop the word clouds for the survey and they are not research participants. The moderator for the Myelopathy Support group is a member of the Steering Group for the Priority Setting Partnership who design and manage the survey. Myelopathy Support is an arm of Myelopathy.org, an international charity for DCM. Myelopathy Support includes an online support group hosted on Facebook (California, USA). The group is closed, and access is moderated by Myelopathy.org volunteers [IS]. Individuals wishing to join the group are required to confirm they have myelopathy and will adhere to community guidelines. Prior experience has demonstrated that demographics and disease characteristics of this group, aside from a female gender predominance, are broadly representative of DCM [18–22].

Over a 2-week period, four posts were pinned to the top of the Myelopathy Support group: (1) “What words do you associate with diagnosis phase of myelopathy?” (2) “What words do you associate with the management phase of myelopathy?” (3) “What words do you associate with the long-term care/living with myelopathy?” (4) “Any other relevant words?”. The posts were accompanied by covering information outlining the background and rationale for this exercise, piloted and approved by IS [Supporting Information 1]. In short, group members were encouraged to add their unrepresented suggestions as comments on the particular post or posts and to vote (as likes or with emotions) the posted words with which they agreed. This exercise was moderated by a group administrator [IS]. No data was collected on individual participants, e.g., their demographics. There was no limit on how many times a group member could contribute.

Following the 2-week period, the submitted words and their respective votes were reviewed by the AO Spine RECODE-DCM Management Group [ODM, ES, DK, IS, BMD, OH] and duplicated words were removed. By mutual agreement, words considered to be out of scope were removed, and words felt to be better reflected in a different section were moved. Through consultation with representatives of the other Healthcare Professional stakeholder group (ES, SS) and discussion amongst the management group, some additional word suggestions were put forward. The aim of this was to include the perspective of an additionally under-represented stakeholder group [23] (other healthcare professionals) while ensuring this remained patient-centered by presenting any suggestions back to the group.

The final lists were then re-posted to the group as a series of polls, to allow members to review the list and vote for the words with which they most agreed [Supporting Information 2]. Users could continue to submit additional suggestions, which were then available to be polled. Users could not vote more than once. This second-round exercise ran for a further 2 weeks. The findings were processed by the management group, to combine duplicates and remove out of scope suggestions. Word clouds were generated using WordArt.com (California, USA) for each major section. The size of the word was proportional to the number of votes it received. Words without a vote were not included. The first iteration of the output was used, without modification.

## Results

**Fig. 1:**
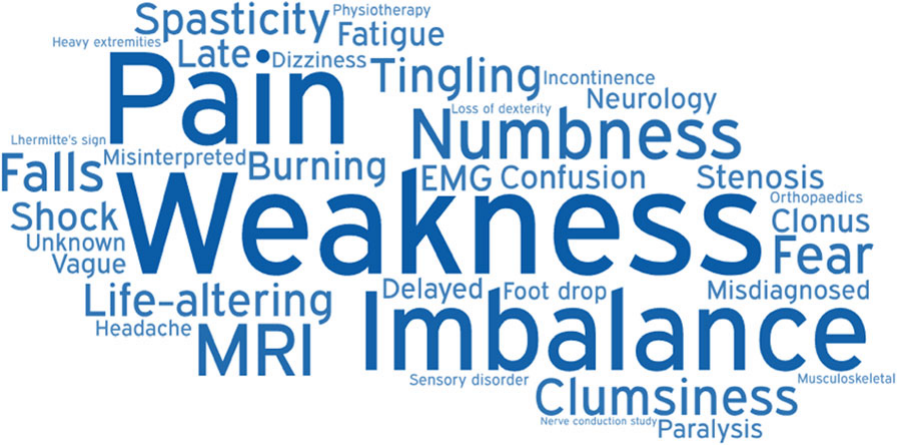
Word cloud for diagnosis

**Fig. 2:**
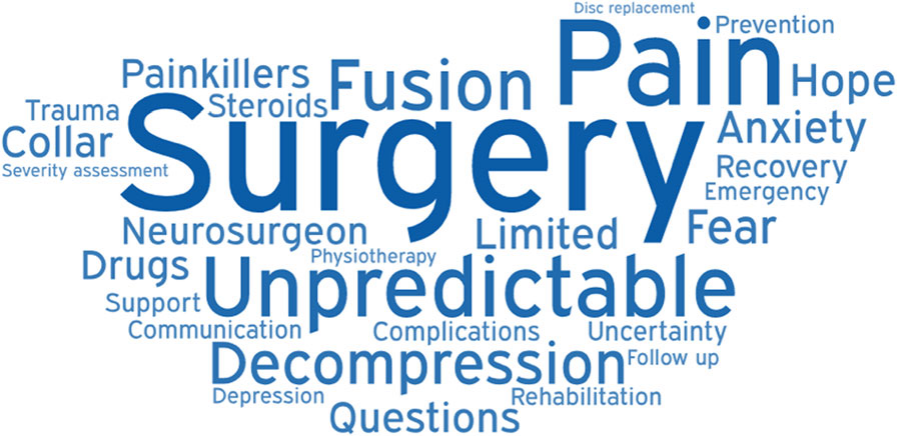
Word cloud for treatment

**Fig. 3:**
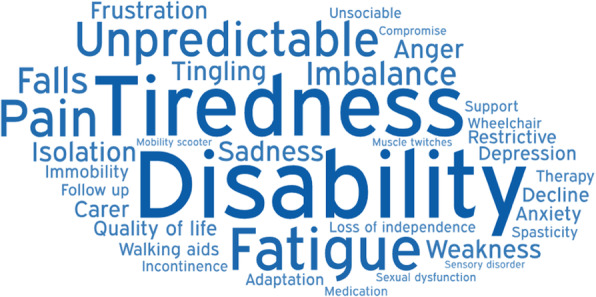
Word cloud for long-term management

**Fig. 4:**
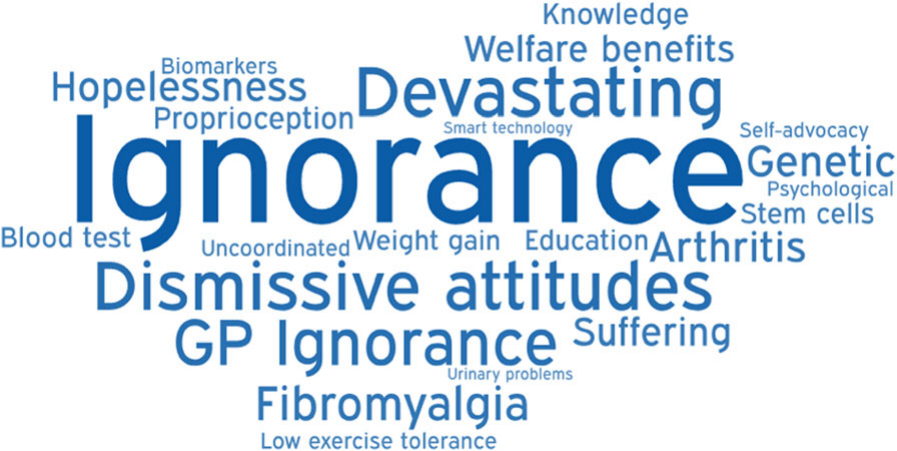
Word cloud for other

Members of the Myelopathy Support online group submitted 79 words, specifically 18 for diagnosis, 16 for treatment, 29 for long-term management, and 16 for other. Following internal discussion, with consultation from ES and SK, a further 25 words were suggested for inclusion, including 4 for diagnosis, 8 for treatment, 8 for long-term management, and 5 for other. It was agreed that “Loss of Vision” was out of scope, and “Depression,” “Walking Problems,” “Worry,” and “Anxiety” were already represented in the long-term management and could be removed from the other category. The final shortlist of words is shown in Table 1 and was placed into Facebook polls as outlined in Supporting Information 3 and Supporting Information 4. In this second round, a further 39 words were added. All words were polled at least once, with “weakness” receiving the maximum votes 47 (Table 2). The data was used to generate word clouds for their respective sections (Figs. 1, 2, 3, and 4).

**Table 1:**
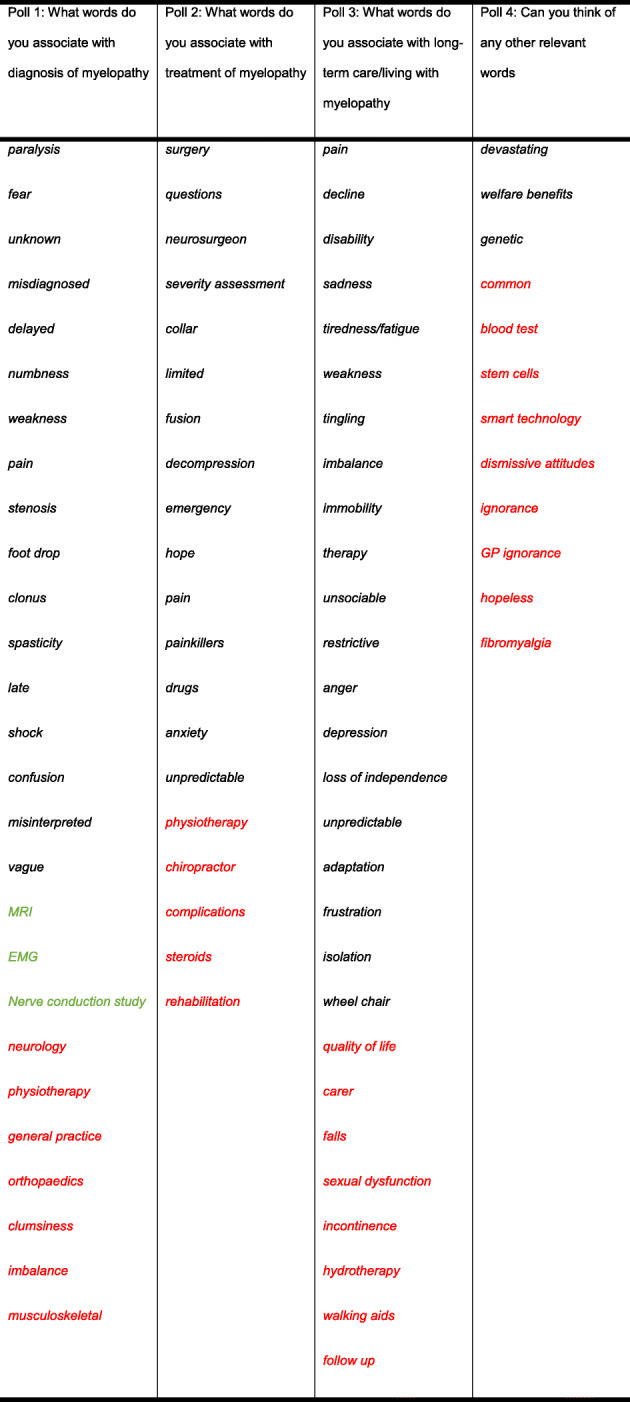
Words and their respective categories, placed into polls for the second stage. Words in green have been moved from treatment to diagnosis. Words in black were submitted by Myelopathy Support members. Words in red were submitted by the management group

**Table 2:**
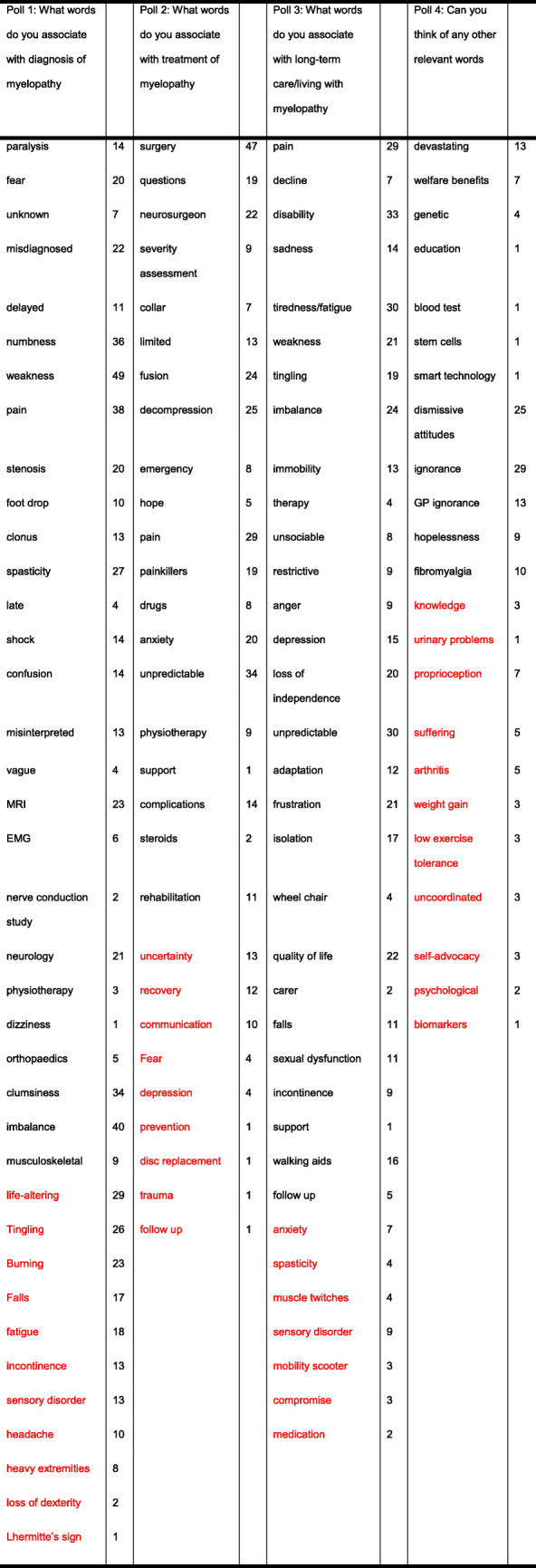
Results of polling. Words submitted as new are in red

## Implementation plan for AO Spine RECODE-DCM

AO Spine RECODE-DCM includes several parallel consensus processes, including a research priority setting partnership and the development of a minimum data set. The AO Spine RECODE-DCM survey will be accessed via a single-entry point, with block randomization to one of the two streams (either Priority Setting Partnership or Minimum Data Set) per stakeholder group (spinal surgeon, other healthcare professionals, persons with DCM and their supporters). This methodology was chosen for efficiency to enable a single recruitment phase. Further detail on the overall process is provided in reference [5].

For participants allocated to the PSP, further 1:1 block randomization will occur to two streams, a survey including word clouds and a second stream without word clouds. For those allocated to the word cloud stream, participants will complete the survey as planned initially. However, after survey completion, respondents will be presented with a brief overview of the development method and aims of the word cloud subproject. Participants will then be presented with each word cloud in turn, with the option to submit further research questions. This approach was taken for two principal reasons. Firstly, to ensure the traditional JLA methodology can be conducted contemporaneously, but still partitioned if required, and secondly, to enable any cross over of responses within the word cloud arm to be considered. Participants will be able to move freely within the survey up until the point they reached a final “thank you” page. Consequently, it will be theoretically possible for participants in the word cloud stream to edit their pre-word cloud form having seen the word clouds. This cannot be tracked by the survey platform. However, by retaining an arm who have no access to the word clouds, any such cross over can be evaluated.

As part of any JLA PSP, the raw suggestions are processed into their individual questions. Then questions deemed “out of scope” are removed, before the remaining questions are collated into common themes, each represented by a summary question. This process is iterative, with a clear audit trail to track where any submission ends up. Consequently, the overall number of questions submitted, alongside the number deemed “in-scope” research questions and the number linked to a summary question appearing in the final research priority list, will be recorded in this evaluation. These numbers will be compared between streams, and for those in the word cloud stream, before and after viewing the word cloud. Metrics will be evaluated overall and per stakeholder group, as well as specifically for each category of question (diagnosis, treatment, long-term management, and other). The impact of the relative size of a word or its relative competition (the number of other words in the cloud) will also be evaluated, by comparing the number of questions generated that match each word and evaluating for a positive correlation. The use of word clouds will be judged meaningful if they prompt a greater breadth of suggestions across the phases of care. Suitable statistical methods will be used for comparisons based on the distribution of the data.

## Discussion

Primarily driven by individuals with DCM, word clouds were developed for associations with the diagnosis, treatment, long-term phases of care, and an “other” category. These will be nested within AO Spine RECODE-DCM, in order to evaluate their role in supporting the research uncertainty gathering phase of a JLA Research Priority Setting Partnership.

The diagnosis word cloud highlights prominent symptoms and MRI, the gold standard diagnostic tool. The treatment section highlighted the gold standard treatment—surgical decompression. The long-term management section highlighted disability and tiredness. The other category highlighted ignorance of the condition. Amongst several pervasive themes were pain and psychological consequences such as anxiety and depression.

The perspective of PwCM has been limited within DCM research to date, but wider experience from healthcare shows this can bring critical insights [8]. In one of the few previous DCM examples, our evaluation of PwCM recovery priorities identified that pain, alongside upper and lower limb function, was most valued [18]. This contrasts the current outcomes of research studies not including PwCM input and supports the rationale for AO Spine RECODE-DCM, and its establishment of a minimum data set. The broad range of ideas displayed goes beyond current research foci [23] indicating the value of engaging PwCM in research design [8].

We hypothesize that this PwCM perspective could be enhanced within a JLA PSP. While PSP were developed to ensure the voice of patients is incorporated into setting a research agenda, this is only really considered by the group as a whole during the final consensus meeting. Put specifically, professionals do not have the opportunity to reflect on “patient” perspectives until summary questions have already been generated and shortlisted. In this nested methodological study, we will therefore evaluate whether the PwCM generated word clouds can help the group as a whole develop research questions and specifically, priority research questions.

Word clouds are a clustering technique mainly applied for the visual analysis of qualitative data. A search of MEDLINE for “Wordcloud” or “Word Cloud,” including title and abstract screening, returned 53 articles (12 August 2019). Articles describe word cloud use to depict survey, workshop/forum, or medical literature data as well as other interesting applications to assist keyword identification for literature searches [24, 25] and to evaluate reference letters for residency programs [26]. There are currently only two references to their use for stimulating ideas and both were deemed effective; first in palliative care, where word clouds were generated by a palliative patient and their family, to support positive memories during the bereavement process [27], and secondly, as part of an education initiative asking medical students to consider “What is professionalism?” [28]. Word cloud usage outside of medicine is much broader [16].

The methodology to form the word clouds was developed by the management group of AO Spine RECODE-DCM. Only a single prior study evaluating the design of word clouds was identified, in which the semantic grouping of word clouds was found to be more effective [15]. In the DCM context, word clouds for each section (diagnosis, treatment, etc.) were derived as opposed to a single word cloud for all DCM concepts. This was chosen to match the predefined questions covering phases of care, and in line with our principal objective to encourage broader research uncertainty gathering. Additionally, a two-stage development process was used, whereby ideas were gathered before being represented to the group for polling. Whether a two-stage development process was required is unclear. It was partly inspired by the two-stage iterative processes that are common to most DELPHI processes. However, it may be more efficient to generate a provisional list internally and then simply develop a poll where users can make additional suggestions. Of note, while unmeasurable, it was our sense that the two-stage process helped to focus the community to the task and its objectives in this instance.

We acknowledge that this was an internet-recruited, convenience sample of persons with DCM. Therefore, the generalization of these word clouds, in the absence of demographics, cannot be fully evaluated. Experience from prior research involving this group indicates their demographics and disease characteristics are broadly representative of DCM [18–22]; moreover, the use of such sampling is the mainstay of traditional JLA PSP [29]. As these details will be captured within AO Spine RECODE-DCM, potential bias can be evaluated.

## Conclusion

We have shown it is feasible to work with PwCM with relevant experience to generate a tool for the AO Spine RECODE-DCM nested methodological study. Once the survey stage is completed, we will be able to evaluate the impact of the word clouds. Further research will be needed to assess the value of any impact in terms of stimulating a more creative research agenda.

## Supplementary Information

**Additional file 1.** Supporting Information 1: Covering information provided to indicate the background and requirements. Supporting Information 2: Covering information for the second round of word cloud development. Supporting Information 3: Words suggested by members of Myelopathy Support, before processing by the RECODE-DCM Management Group. Supporting Information 4: Screen shots from the polls in action [A] Diagnosis and [B] Treatment.

## Data Availability

The datasets used and/or analyzed during the current study are available from the corresponding author on reasonable request.
