# Supplementary material for: The development of lived experience-centered word clouds to support research uncertainty gathering in degenerative cervical myelopathy: results from an engagement process and protocol for their evaluation, via a nested randomized controlled trial
Source: Trials. 2021 Jun 25;22:415. doi: 10.1186/s13063-021-05349-8 (PMC8235822; doi:10.1186/s13063-021-05349-8)
Supplement: Supplementary file 1 — Additional file 1. Supporting Information 1: Covering information provided to indicate the background and requirements. Supporting Information 2: Covering information for the second round of word cloud development. Supporting Information 3: Words suggested by members of Myelopathy Support, before processing by the RECODE-DCM Management Group. Supporting Information 4: Screen shots from the polls in action [A] Diagnosis and [B] Treatment. [file 13063_2021_5349_MOESM1_ESM.docx]

# Supporting Information

| Myelopathy.org is supporting an international consultation process, funded by AO Spine (https://aospine.aofoundation.org), to create a list of the most important questions that need answering by DCM. This is supported by the JLA, who oversee this process. (<http://www.jla.nihr.ac.uk/>), which they advise as a consultation does not require formal ethical approval. The long-term ambition is to make sure research is being conducted that matters to you.  In due course, you will all be invited to share your thoughts, but the survey is not quite ready yet. In fact the investigators are currently designing the opening survey, and are looking at ways to help people come up with research questions - as you can imagine, it is difficult to think of ideas with a big general question, and therefore prompts can be helpful to stimulate ideas. They would like to use word clouds to do this, but only want words in the word cloud that matter to those affected by DCM. That’s why we need your help. All the words that you suggest will be included, and the number of positive interactions with a word will change how large it appears in the word cloud. |
| --- |

Supporting Information 1: Covering information provided to indicate the background and requirements.

| 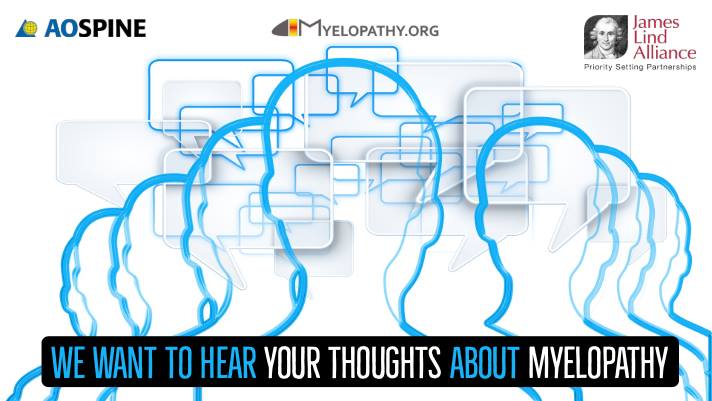  Myelopathy.org is working with AOSpine and the James Lind Alliance to create a list of the most importance questions that need answering in degenerative cervical myelopathy.  As our first step we asked what words you associate with myelopathy. The objective is for these words to stimulate research ideas. Thank you for sharing your words on our posts on the past few weeks. The next stage is now to find out which words are the most important. This information will be used to create word clouds, with the size of the word in the word cloud related to how popular that word is in your voting.  We have created four Facebook polls, which you should see below this post. Please vote for every word that you associate with myelopathy and add in words that are not already on this list so that other people can vote on them!  Thank you for supporting us as we work towards improving the lives of those with degenerative cervical myelopathy. Whatever your connection to myelopathy, we want to hear from you.  [#cervicalmyelopathy](https://www.facebook.com/hashtag/cervicalmyelopathy?source=feed_text&epa=HASHTAG&__xts__%5B0%5D=68.ARDDId0223J5ZWEZTwgFedXvyKER2AFvYRxfAmGmTRVaOYfSt6_2BdV-xAJKNzOib41LJ3JAs8x1OX_tycjp2WXUiXy-RNFMVBekho9BiTTP_bcf2juK54oLzBN5EaiuiSKv1vXxhSmXxKzyoMcfxOv4xt4xsR2yn6fOx1AZ1C1xUGvYZWNJcq7EXcdTA5Zn_CR62Hhq8inkUVIMcLqzFGRKptTZqZWDpO9BcWHjllTBgvRmPP93pidC4EVgELcxC_UUzkNBCOt9Un7UYJQvogJPOgOjhcjK0s0bIZnv8gSVsnzcDjK6addl1JbfZF1w1omD5F3j4p3MMfY6UT_NtOpQm8r7ayvL8brtNNsFoY1fL1t9oWoS4Z-6RzCjwHt6yjEGVKbSj9qJZL_TqGnHjyKDMLGuJf7qnvL3j4tlgL79nSixtzqmKf4WO6JOsq_uwFVBjA0HvBuRMcxoaIfamWk6E2MT2ykno-tnWJbkh2nIOOZfdHwIeizcVP62Og&__tn__=%2ANKH-R) [#aospine](https://www.facebook.com/hashtag/aospine?source=feed_text&epa=HASHTAG&__xts__%5B0%5D=68.ARDDId0223J5ZWEZTwgFedXvyKER2AFvYRxfAmGmTRVaOYfSt6_2BdV-xAJKNzOib41LJ3JAs8x1OX_tycjp2WXUiXy-RNFMVBekho9BiTTP_bcf2juK54oLzBN5EaiuiSKv1vXxhSmXxKzyoMcfxOv4xt4xsR2yn6fOx1AZ1C1xUGvYZWNJcq7EXcdTA5Zn_CR62Hhq8inkUVIMcLqzFGRKptTZqZWDpO9BcWHjllTBgvRmPP93pidC4EVgELcxC_UUzkNBCOt9Un7UYJQvogJPOgOjhcjK0s0bIZnv8gSVsnzcDjK6addl1JbfZF1w1omD5F3j4p3MMfY6UT_NtOpQm8r7ayvL8brtNNsFoY1fL1t9oWoS4Z-6RzCjwHt6yjEGVKbSj9qJZL_TqGnHjyKDMLGuJf7qnvL3j4tlgL79nSixtzqmKf4WO6JOsq_uwFVBjA0HvBuRMcxoaIfamWk6E2MT2ykno-tnWJbkh2nIOOZfdHwIeizcVP62Og&__tn__=%2ANKH-R) [#myelopathy](https://www.facebook.com/hashtag/myelopathy?source=feed_text&epa=HASHTAG&__xts__%5B0%5D=68.ARDDId0223J5ZWEZTwgFedXvyKER2AFvYRxfAmGmTRVaOYfSt6_2BdV-xAJKNzOib41LJ3JAs8x1OX_tycjp2WXUiXy-RNFMVBekho9BiTTP_bcf2juK54oLzBN5EaiuiSKv1vXxhSmXxKzyoMcfxOv4xt4xsR2yn6fOx1AZ1C1xUGvYZWNJcq7EXcdTA5Zn_CR62Hhq8inkUVIMcLqzFGRKptTZqZWDpO9BcWHjllTBgvRmPP93pidC4EVgELcxC_UUzkNBCOt9Un7UYJQvogJPOgOjhcjK0s0bIZnv8gSVsnzcDjK6addl1JbfZF1w1omD5F3j4p3MMfY6UT_NtOpQm8r7ayvL8brtNNsFoY1fL1t9oWoS4Z-6RzCjwHt6yjEGVKbSj9qJZL_TqGnHjyKDMLGuJf7qnvL3j4tlgL79nSixtzqmKf4WO6JOsq_uwFVBjA0HvBuRMcxoaIfamWk6E2MT2ykno-tnWJbkh2nIOOZfdHwIeizcVP62Og&__tn__=%2ANKH-R) [#jla](https://www.facebook.com/hashtag/jla?source=feed_text&epa=HASHTAG&__xts__%5B0%5D=68.ARDDId0223J5ZWEZTwgFedXvyKER2AFvYRxfAmGmTRVaOYfSt6_2BdV-xAJKNzOib41LJ3JAs8x1OX_tycjp2WXUiXy-RNFMVBekho9BiTTP_bcf2juK54oLzBN5EaiuiSKv1vXxhSmXxKzyoMcfxOv4xt4xsR2yn6fOx1AZ1C1xUGvYZWNJcq7EXcdTA5Zn_CR62Hhq8inkUVIMcLqzFGRKptTZqZWDpO9BcWHjllTBgvRmPP93pidC4EVgELcxC_UUzkNBCOt9Un7UYJQvogJPOgOjhcjK0s0bIZnv8gSVsnzcDjK6addl1JbfZF1w1omD5F3j4p3MMfY6UT_NtOpQm8r7ayvL8brtNNsFoY1fL1t9oWoS4Z-6RzCjwHt6yjEGVKbSj9qJZL_TqGnHjyKDMLGuJf7qnvL3j4tlgL79nSixtzqmKf4WO6JOsq_uwFVBjA0HvBuRMcxoaIfamWk6E2MT2ykno-tnWJbkh2nIOOZfdHwIeizcVP62Og&__tn__=%2ANKH-R) [#myelopathymatters](https://www.facebook.com/hashtag/myelopathymatters?source=feed_text&epa=HASHTAG&__xts__%5B0%5D=68.ARDDId0223J5ZWEZTwgFedXvyKER2AFvYRxfAmGmTRVaOYfSt6_2BdV-xAJKNzOib41LJ3JAs8x1OX_tycjp2WXUiXy-RNFMVBekho9BiTTP_bcf2juK54oLzBN5EaiuiSKv1vXxhSmXxKzyoMcfxOv4xt4xsR2yn6fOx1AZ1C1xUGvYZWNJcq7EXcdTA5Zn_CR62Hhq8inkUVIMcLqzFGRKptTZqZWDpO9BcWHjllTBgvRmPP93pidC4EVgELcxC_UUzkNBCOt9Un7UYJQvogJPOgOjhcjK0s0bIZnv8gSVsnzcDjK6addl1JbfZF1w1omD5F3j4p3MMfY6UT_NtOpQm8r7ayvL8brtNNsFoY1fL1t9oWoS4Z-6RzCjwHt6yjEGVKbSj9qJZL_TqGnHjyKDMLGuJf7qnvL3j4tlgL79nSixtzqmKf4WO6JOsq_uwFVBjA0HvBuRMcxoaIfamWk6E2MT2ykno-tnWJbkh2nIOOZfdHwIeizcVP62Og&__tn__=%2ANKH-R)[#myelopathysurvey](https://www.facebook.com/hashtag/myelopathysurvey?source=feed_text&epa=HASHTAG&__xts__%5B0%5D=68.ARDDId0223J5ZWEZTwgFedXvyKER2AFvYRxfAmGmTRVaOYfSt6_2BdV-xAJKNzOib41LJ3JAs8x1OX_tycjp2WXUiXy-RNFMVBekho9BiTTP_bcf2juK54oLzBN5EaiuiSKv1vXxhSmXxKzyoMcfxOv4xt4xsR2yn6fOx1AZ1C1xUGvYZWNJcq7EXcdTA5Zn_CR62Hhq8inkUVIMcLqzFGRKptTZqZWDpO9BcWHjllTBgvRmPP93pidC4EVgELcxC_UUzkNBCOt9Un7UYJQvogJPOgOjhcjK0s0bIZnv8gSVsnzcDjK6addl1JbfZF1w1omD5F3j4p3MMfY6UT_NtOpQm8r7ayvL8brtNNsFoY1fL1t9oWoS4Z-6RzCjwHt6yjEGVKbSj9qJZL_TqGnHjyKDMLGuJf7qnvL3j4tlgL79nSixtzqmKf4WO6JOsq_uwFVBjA0HvBuRMcxoaIfamWk6E2MT2ykno-tnWJbkh2nIOOZfdHwIeizcVP62Og&__tn__=%2ANKH-R) |
| --- |

Supporting Information 2: Covering information for the second round of word cloud development.

| What words do you associate with diagnosis of myelopathy | What words do you associate with treatment of myelopathy | What words do you associate with long-term care/living with myelopathy | Can you think of any other relevant words |
| --- | --- | --- | --- |
| paralysis | surgery | pain | devastating |
| disability | questions | decline | pain |
| fear | MRI | disabled | isolation |
| unknown | EMG | fatigue | worry |
| misdiagnosed | nerve conduction study | sadness | genetic |
| delayed | disappointing | tiredness | walking problems |
| numbness | fusion | poor vision | sad |
| weakness | decompression | weakness | loss |
| pain | emergency | tingling | depression |
| stenosis | hope | sensitivity | anxiety |
| foot drop | pain | crying | fear |
| clonus | painkillers | imbalance | dismissive attitudes |
| spasticity | drugs | immobility | ignorance |
| late | limitations | therapy | GP ignorance |
| shock | anxiety | unsociable | hopeless |
| confusion | unpredictable | grief | exhaustion |
| misinterpreted |  | restrictive |  |
| vague |  | anger |  |
|  |  | depression |  |
|  |  | loss of independence |  |
|  |  | adaptation |  |
|  |  | frustration |  |
|  |  | isolation |  |
|  |  | dependence |  |
|  |  | wheel chair |  |
|  |  | struggle |  |

Supporting Information 3: Words suggested by members of Myelopathy Support, before processing by the RECODE-DCM Management Group.

A

B


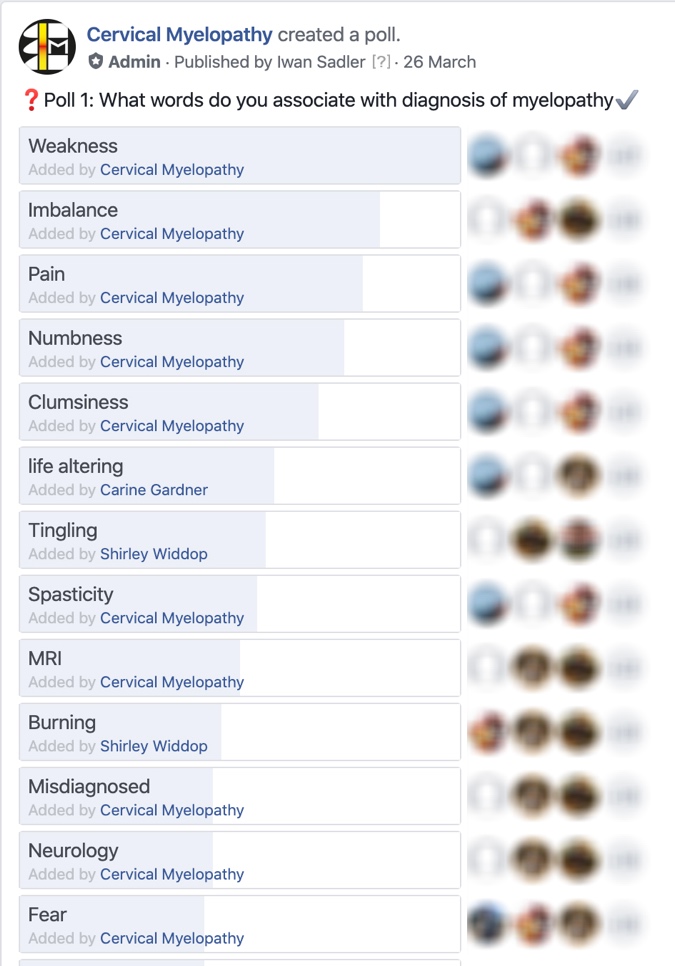

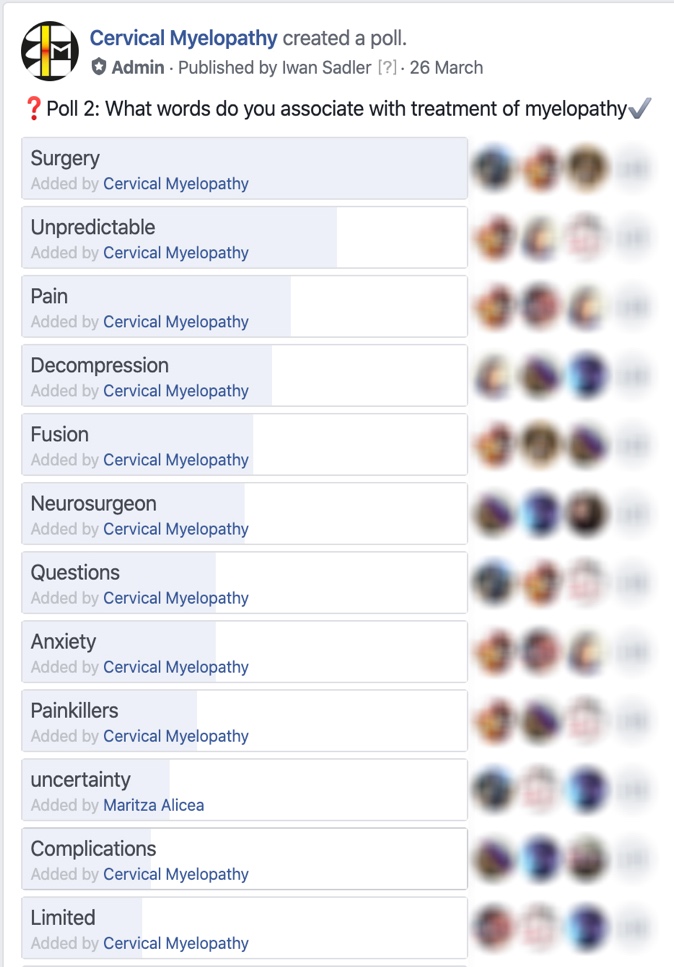


Supporting Information 4: Screen shots from the polls in action [A] Diagnosis and [B] Treatment.
